# Crystal structure of 3-(4-chloro­phen­oxy)-4-(2-nitro­phen­yl)azetidin-2-one with an unknown solvate

**DOI:** 10.1107/S2056989014025845

**Published:** 2015-01-01

**Authors:** Sevim Türktekin Çelikesir, Mehmet Akkurt, Aliasghar Jarrahpour, Habib Allah Shafie, Ömer Çelik

**Affiliations:** a Department of Physics, Faculty of Sciences, Erciyes University, 38039 Kayseri, Turkey; b Department of Chemistry, College of Sciences, Shiraz University, 71454 Shiraz, Iran; c Department of Physics, Faculty of Education, Dicle University, 21280, Diyarbakir, Turkey, and, Science and Technology Application and Research Center, Dicle University, 21280, Diyarbakir, Turkey

**Keywords:** crystal structure, β-lactam ring, *C*(4) chain, hydrogen bonding, N-unsubstituted 2-azetidinone, hydrogen bonds, C—H⋯π inter­actions

## Abstract

In the title compound, C_15_H_11_ClN_2_O_4_, the central β-lactam ring is approximately planar [maximum deviation = 0.044 (2) Å for the N atom from the mean plane] and subtends dihedral angles of 61.17 (11) and 40.21 (12) °, respectively, with the nitro and chloro­benzene rings. Both substituents lie to the same side of the β-lactam core. In the crystal, N—H⋯O hydrogen bonds link the mol­ecules into *C*(4) chains propagating in [010]. The chains are cross-linked by C—H⋯O and weak C—H⋯π inter­actions, generating a three-dimensional network. The solvent mol­ecules were found to be highly disordered and their contribution to the scattering was removed with the SQUEEZE procedure in *PLATON* [Spek (2009 ▸). *Acta Cryst.* D**65**, 148–155], which indicated a solvent cavity of volume 318 Å^3^ containing approximately 114 electrons. These solvent mol­ecules are not considered in the given chemical formula and other crystal data.

## Related literature   

For the application of N-unsubstituted 2-azetidinones in the synthesis of β-lactam anti­biotics, see: Cossio *et al.* (1987 ▸); Jarrahpour & Zarei (2007 ▸, 2008 ▸). For a related structure with a β-lactam ring, see: Butcher *et al.* (2011 ▸).
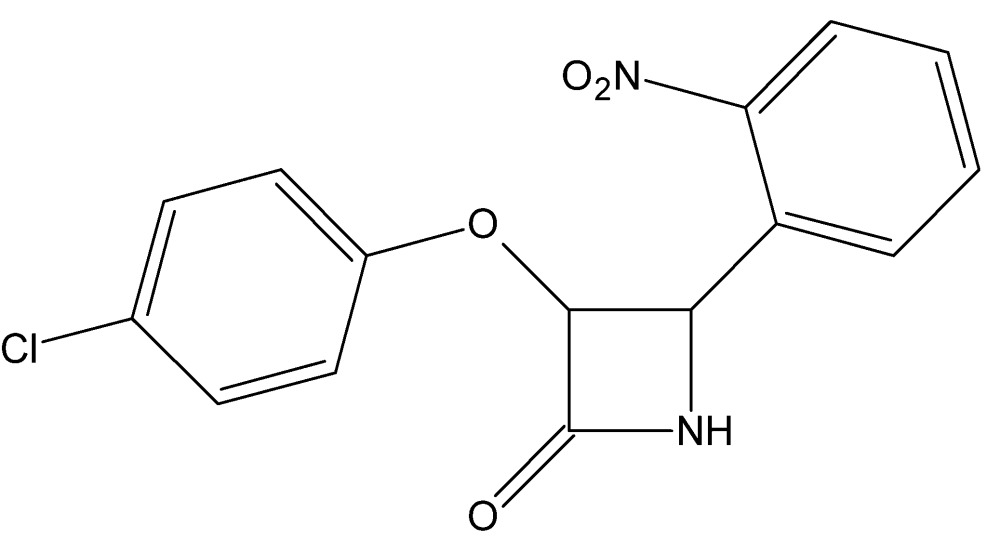


## Experimental   

### Crystal data  

C_15_H_11_ClN_2_O_4_
*M* *_r_* = 318.71
Monoclinic, 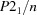

*a* = 16.9505 (4) Å
*b* = 4.6517 (1) Å
*c* = 21.7167 (6) Å
β = 99.757 (1)°
*V* = 1687.56 (7) Å^3^
*Z* = 4
Mo *K*α radiation
μ = 0.24 mm^−1^
*T* = 296 K
0.30 × 0.20 × 0.15 mm

### Data collection  

Bruker APEXII CCD diffractometer
16293 measured reflections
4358 independent reflections
3158 reflections with *I* > 2σ(*I*)
*R*_int_ = 0.022

### Refinement  

*R*[*F* ^2^ > 2σ(*F* ^2^)] = 0.049
*wR*(*F* ^2^) = 0.135
*S* = 1.04
4358 reflections
199 parameters
H-atom parameters constrained
Δρ_max_ = 0.35 e Å^−3^
Δρ_min_ = −0.23 e Å^−3^

### 

Data collection: *APEX2* (Bruker, 2007 ▸); cell refinement: *SAINT* (Bruker, 2007 ▸); data reduction: *SAINT*; program(s) used to solve structure: *SHELXS2014* (Sheldrick, 2008 ▸); program(s) used to refine structure: *SHELXL2014* (Sheldrick, 2008 ▸); molecular graphics: *ORTEP-3 for Windows* (Farrugia, 2012 ▸); software used to prepare material for publication: *PLATON* (Spek, 2009 ▸).

## Supplementary Material

Crystal structure: contains datablock(s) global, I. DOI: 10.1107/S2056989014025845/hb7322sup1.cif

Structure factors: contains datablock(s) I. DOI: 10.1107/S2056989014025845/hb7322Isup2.hkl

Supporting information file. DOI: 10.1107/S2056989014025845/hb7322Isup3.cml

**Fig. 1.**
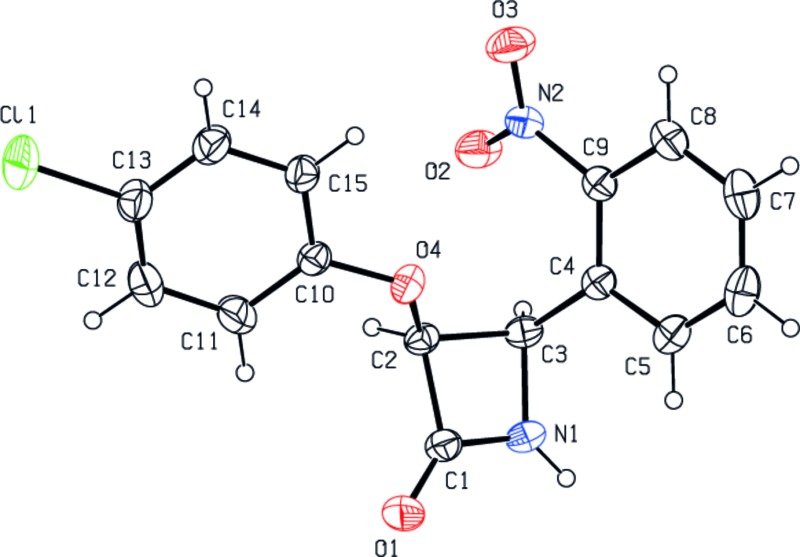
View of the title compound with displacement ellipsoids for non-H atoms drawn at the 30% probability level.

**Fig. 2.**
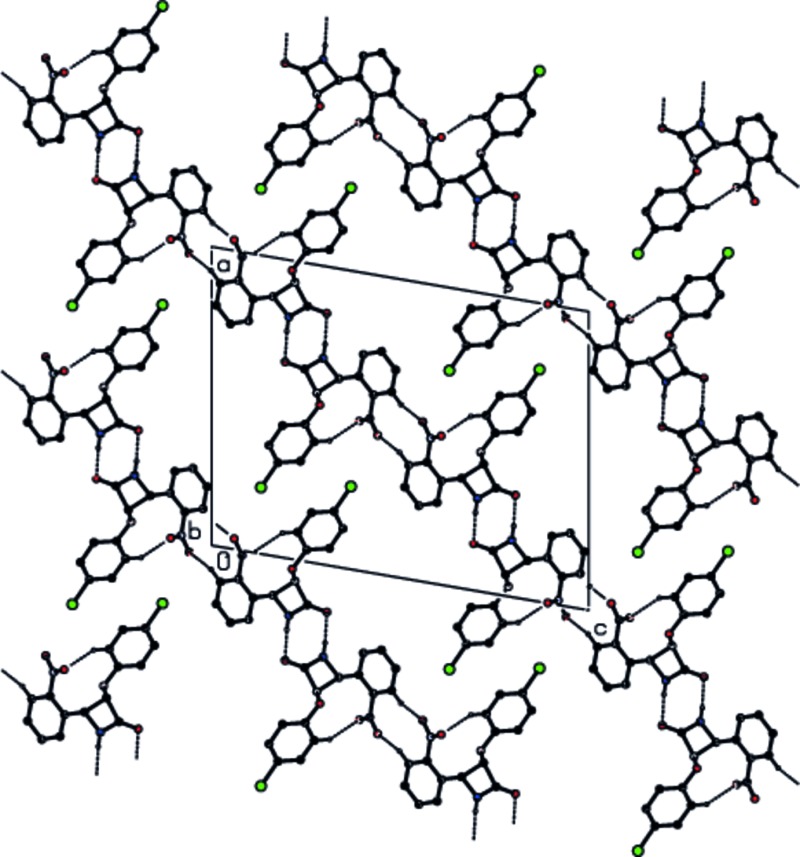
View of the hydrogen bonding of the title compound along b axis. Only H atoms involved in H bonding are shown.

. DOI: 10.1107/S2056989014025845/hb7322fig1.tif
View of the title compound with displacement ellipsoids for non-H atoms drawn at the 30% probability level.

b . DOI: 10.1107/S2056989014025845/hb7322fig2.tif
View of the hydrogen bonding of the title compound along *b* axis. Only H atoms involved in H bonding are shown.

CCDC reference: 1036035

Additional supporting information:  crystallographic information; 3D view; checkCIF report

## Figures and Tables

**Table 1:** Hydrogen-bond geometry (, ) *Cg*2 is the centroid of the nitrobenzene ring (C4C9).

*D*H*A*	*D*H	H*A*	*D* *A*	*D*H*A*
N1H1O1^i^	0.86	2.09	2.936(2)	166
C8H8O3^ii^	0.93	2.53	3.307(2)	142
C15H15O2^iii^	0.93	2.51	3.338(2)	149
C3H3*Cg*2^iv^	0.98	2.70	3.5118(19)	141

Symmetry codes: (i) 
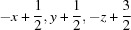
; (ii) 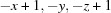; (iii) 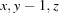; (iv) 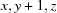.

## supplementary crystallographic information

### S1. Comment

N-Unsubstituted 2-azetidinones offer major synthetic opportunities in the synthesis of β-lactam antibiotics such as the carbapenems, penams, monobactams, nocardicins, and the glutamine synthetase inhibitor, tabtoxin (Cossio, *et al.*, 1987) The application of N-unsubstituted 2-azetidinones in the semi-synthesis of the anticancer agents, Taxol and Taxotere has also been reported (Jarrahpour, *et al.*, 2007). N-Unsubstituted 2-azetidinones have been prepared by several methods. Among these methods, oxidative cleavage by ceric ammonium nitrate (CAN) of *p*-alkoxyphenyl moiety attached to the nitrogen of the β-lactam ring offers the most direct synthesis of N-unsubstituted β-lactams (Jarrahpour, *et al.*, 2008).

In the title compound (Fig. 1), the β-lactam ring is nearly planar with a maximum deviation of 0.044 (2) Å for N1 from the mean plane. The carboxyl O atom O1 attached to the β-lactam ring deviates by -0.137 (1) Å from the mean plane of the ring. The β-lactam ring makes dihedral angles of 61.17 (11) and 40.21 (12) ° with the nitro and choloro-benzene rings, respectively.

All bond lengths and angles are comparable with those reported in a related structure (Butcher *et al.*, 2011).

In the crystal, molecules are linked into [010] *C*(4) chains by N—H···O hydrogen bonds. C—H···O hydrogen bonds (Fig. 2) and weak C—H···π interactions link the chains into a three-dimensional network (Table 1).

### S2. Experimental

A solution of (NH_4_)_2_Ce(NO_3_)_6_ (CAN) (3.00 mmol) in water (15.00 ml) was added dropwise to a solution of β-lactam (1.00 mmol) in CH_3_CN (30.00 ml) at room temperature and stirred for 45 min. Then water (30.00 ml) was added and the mixture was extracted with EtOAc (3 × 20 ml) and washed with 10% of NaHCO_3_ (40 ml). The aqueous layer of NaHCO_3_ was extracted again with EtOAc (15 ml), and all organic extracts were combined and washed successively with 10% NaHSO_3_ (2×30 ml), 10% NaHCO_3_ (20 ml), brine (20 ml) and then dried over sodium sulfate. After filtration and evaporation of the solvent in vacuo, the crude product was purified by column chromatography or recrystallization from hexane/EtOAc (4:6) solution to give colourless prisms (Yield 75%). Mp 350–352 K IR (KBr, cm^-1^): 1774 (CO β-lactam), 3217 (NH). ^1^H NMR (250 MHz, DMSO-d_6_) δ(p.p.m.): 5.74 (H-3, d, 1H, J=5.5 Hz), 6.23 (H-4, d, 1H, J=5.5 Hz), 7.27–8.15 (aromatic H, m, 8H), 9.13 (NH, brs, 1H). ^13^C NMR (62.9 MHz, DMSO-d_6_) δ(p.p.m.): 54.60 (C-4), 83.18 (C-3), 117.5, 124.7, 125.9, 128.7, 128.9, 129.1, 132.8, 134.4, 147.4, 155.8 (aromatic carbons), 165.90 (CO, β- lactam). Analysis calculated for C_15_H_11_ClN_2_O_4_: C, 51.01; H, 2.85; N, 7.93%. Found: C, 51.03; H, 2.87; N, 7.91%.

### S3. Refinement

C and N-bound H atoms were positioned geometrically (C—H = 0.93 - 0.98 Å and N—H =0.86 Å), and refined using a riding model with *U*_iso_(H) = 1.2*U*_eq_(C,*N*). The twenty two reflections were omitted owing to bad disagreement. The crystal quality and data was not good enough. A region of disordered electron density, most probably disordered solvent molecules, occupying voids of *ca* 318 Å^3^ for an electron count of 114, was removed with the SQUEEZE procedure in *PLATON* [Spek (2009). Acta Cryst. D65, 148–155] following unsuccessful attempts to model it as a plausible solvent molecule. Their formula mass and unit-cell characteristics were not taken into account during refinement.

### Crystal data

**Table:**

C_15_H_11_ClN_2_O_4_	*F*(000) = 656
*M**_r_* = 318.71	*D*_x_ = 1.254 Mg m^−^^3^
Monoclinic, *P*2_1_/*n*	Mo *K*α radiation, λ = 0.71073 Å
Hall symbol: -P 2yn	Cell parameters from 6230 reflections
*a* = 16.9505 (4) Å	θ = 2.4–28.7°
*b* = 4.6517 (1) Å	µ = 0.24 mm^−^^1^
*c* = 21.7167 (6) Å	*T* = 296 K
β = 99.757 (1)°	Prism, colourless
*V* = 1687.56 (7) Å^3^	0.30 × 0.20 × 0.15 mm
*Z* = 4	

### Data collection

**Table:**

Bruker APEXII CCD diffractometer	3158 reflections with *I* > 2σ(*I*)
Radiation source: sealed tube	*R*_int_ = 0.022
Graphite monochromator	θ_max_ = 28.8°, θ_min_ = 1.7°
φ and ω scans	*h* = −21→22
16293 measured reflections	*k* = −6→6
4358 independent reflections	*l* = −28→29

### Refinement

**Table:**

Refinement on *F*^2^	0 restraints
Least-squares matrix: full	Hydrogen site location: inferred from neighbouring sites
*R*[*F*^2^ > 2σ(*F*^2^)] = 0.049	H-atom parameters constrained
*wR*(*F*^2^) = 0.135	*w* = 1/[σ^2^(*F*_o_^2^) + (0.0607*P*)^2^ + 0.3362*P*] where *P* = (*F*_o_^2^ + 2*F*_c_^2^)/3
*S* = 1.04	(Δ/σ)_max_ = 0.001
4358 reflections	Δρ_max_ = 0.35 e Å^−^^3^
199 parameters	Δρ_min_ = −0.23 e Å^−^^3^

### Special details

**Table:**

Geometry. Bond distances, angles *etc*. have been calculated using the rounded fractional coordinates. All su's are estimated from the variances of the (full) variance-covariance matrix. The cell e.s.d.'s are taken into account in the estimation of distances, angles and torsion angles
Refinement. Refinement on *F*^2^ for ALL reflections except those flagged by the user for potential systematic errors. Weighted *R*-factors *wR* and all goodnesses of fit *S* are based on *F*^2^, conventional *R*-factors *R* are based on *F*, with *F* set to zero for negative *F*^2^. The observed criterion of *F*^2^ > σ(*F*^2^) is used only for calculating -*R*-factor-obs *etc*. and is not relevant to the choice of reflections for refinement. *R*-factors based on *F*^2^ are statistically about twice as large as those based on *F*, and *R*-factors based on ALL data will be even larger.

### Fractional atomic coordinates and isotropic or equivalent isotropic displacement parameters (Å^2^)

**Table:**

	*x*	*y*	*z*	*U*_iso_*/*U*_eq_	
Cl1	0.77735 (3)	0.02466 (16)	0.86934 (3)	0.0896 (2)	
O1	0.34325 (8)	0.4694 (3)	0.80529 (6)	0.0684 (5)	
O2	0.49880 (7)	0.6846 (3)	0.60857 (6)	0.0615 (5)	
O3	0.53550 (8)	0.3225 (3)	0.55969 (6)	0.0686 (5)	
O4	0.46717 (6)	0.2832 (2)	0.71337 (5)	0.0437 (3)	
N1	0.30955 (8)	0.6612 (4)	0.70465 (6)	0.0523 (5)	
N2	0.48444 (8)	0.4599 (3)	0.58069 (6)	0.0440 (4)	
C1	0.35588 (9)	0.5438 (4)	0.75408 (7)	0.0462 (5)	
C2	0.42874 (8)	0.5444 (3)	0.72098 (7)	0.0374 (4)	
C3	0.36874 (9)	0.6469 (4)	0.66256 (7)	0.0399 (5)	
C4	0.34857 (9)	0.4352 (3)	0.60981 (7)	0.0377 (4)	
C5	0.27331 (10)	0.3099 (4)	0.59775 (8)	0.0503 (5)	
C6	0.25251 (11)	0.1159 (5)	0.54958 (9)	0.0632 (7)	
C7	0.30631 (13)	0.0396 (4)	0.51201 (9)	0.0625 (7)	
C8	0.38224 (11)	0.1547 (4)	0.52289 (7)	0.0509 (6)	
C9	0.40248 (9)	0.3484 (3)	0.57097 (7)	0.0387 (5)	
C10	0.54003 (8)	0.2358 (3)	0.75211 (7)	0.0387 (4)	
C11	0.55796 (12)	0.3387 (5)	0.81220 (8)	0.0661 (7)	
C12	0.63188 (13)	0.2760 (6)	0.84797 (8)	0.0733 (8)	
C13	0.68458 (10)	0.1055 (5)	0.82418 (8)	0.0559 (6)	
C14	0.66690 (10)	0.0022 (4)	0.76469 (9)	0.0555 (6)	
C15	0.59401 (9)	0.0697 (4)	0.72819 (8)	0.0470 (5)	
H1	0.26120	0.72420	0.69920	0.0630*	
H2	0.46700	0.69550	0.73710	0.0450*	
H3	0.38230	0.83720	0.64800	0.0480*	
H5	0.23580	0.35770	0.62280	0.0600*	
H6	0.20140	0.03640	0.54260	0.0760*	
H7	0.29160	−0.08920	0.47930	0.0750*	
H8	0.41950	0.10220	0.49800	0.0610*	
H11	0.52090	0.44930	0.82870	0.0790*	
H12	0.64530	0.35050	0.88810	0.0880*	
H14	0.70350	−0.11280	0.74870	0.0670*	
H15	0.58190	0.00190	0.68740	0.0560*	

### Atomic displacement parameters (Å^2^)

**Table:**

	*U*^11^	*U*^22^	*U*^33^	*U*^12^	*U*^13^	*U*^23^
Cl1	0.0624 (3)	0.1234 (6)	0.0723 (3)	0.0190 (3)	−0.0193 (3)	0.0066 (3)
O1	0.0550 (7)	0.1050 (12)	0.0494 (7)	−0.0149 (7)	0.0209 (6)	0.0013 (7)
O2	0.0577 (7)	0.0584 (9)	0.0733 (8)	−0.0162 (6)	0.0251 (6)	−0.0204 (7)
O3	0.0539 (7)	0.0755 (10)	0.0824 (9)	0.0055 (7)	0.0291 (7)	−0.0164 (8)
O4	0.0356 (5)	0.0379 (7)	0.0547 (6)	0.0017 (5)	−0.0002 (4)	−0.0061 (5)
N1	0.0371 (7)	0.0678 (11)	0.0547 (8)	0.0115 (7)	0.0153 (6)	−0.0048 (7)
N2	0.0467 (7)	0.0474 (9)	0.0407 (6)	−0.0008 (6)	0.0152 (5)	0.0008 (6)
C1	0.0378 (8)	0.0556 (11)	0.0471 (8)	−0.0065 (7)	0.0130 (6)	−0.0086 (8)
C2	0.0338 (7)	0.0349 (9)	0.0448 (7)	−0.0034 (6)	0.0107 (6)	−0.0054 (6)
C3	0.0388 (7)	0.0364 (9)	0.0468 (8)	0.0046 (6)	0.0139 (6)	0.0006 (7)
C4	0.0385 (7)	0.0336 (8)	0.0408 (7)	0.0029 (6)	0.0058 (6)	0.0060 (6)
C5	0.0383 (8)	0.0536 (11)	0.0582 (9)	0.0017 (7)	0.0057 (7)	0.0038 (8)
C6	0.0496 (10)	0.0607 (13)	0.0722 (12)	−0.0118 (9)	−0.0102 (9)	0.0016 (10)
C7	0.0732 (13)	0.0551 (12)	0.0522 (9)	−0.0058 (10)	−0.0093 (9)	−0.0082 (9)
C8	0.0623 (10)	0.0483 (11)	0.0408 (8)	0.0036 (9)	0.0051 (7)	−0.0030 (7)
C9	0.0431 (8)	0.0354 (9)	0.0375 (7)	0.0016 (7)	0.0065 (6)	0.0032 (6)
C10	0.0348 (7)	0.0390 (9)	0.0424 (7)	−0.0039 (6)	0.0069 (6)	0.0008 (6)
C11	0.0613 (11)	0.0918 (17)	0.0446 (9)	0.0206 (11)	0.0075 (8)	−0.0113 (10)
C12	0.0692 (12)	0.1051 (19)	0.0405 (9)	0.0120 (12)	−0.0056 (8)	−0.0085 (10)
C13	0.0451 (9)	0.0680 (13)	0.0511 (9)	0.0025 (9)	−0.0021 (7)	0.0074 (9)
C14	0.0402 (8)	0.0628 (13)	0.0621 (10)	0.0075 (8)	0.0050 (7)	−0.0067 (9)
C15	0.0396 (8)	0.0516 (11)	0.0481 (8)	−0.0014 (7)	0.0030 (6)	−0.0101 (8)

### Geometric parameters (Å, º)

**Table:**

Cl1—C13	1.7474 (18)	C8—C9	1.378 (2)
O1—C1	1.218 (2)	C10—C15	1.366 (2)
O2—N2	1.2118 (19)	C10—C11	1.375 (2)
O3—N2	1.2245 (19)	C11—C12	1.389 (3)
O4—C2	1.4016 (17)	C12—C13	1.360 (3)
O4—C10	1.3891 (18)	C13—C14	1.363 (3)
N1—C1	1.335 (2)	C14—C15	1.386 (2)
N1—C3	1.469 (2)	C2—H2	0.9800
N2—C9	1.464 (2)	C3—H3	0.9800
C1—C2	1.531 (2)	C5—H5	0.9300
N1—H1	0.8600	C6—H6	0.9300
C2—C3	1.560 (2)	C7—H7	0.9300
C3—C4	1.505 (2)	C8—H8	0.9300
C4—C5	1.387 (2)	C11—H11	0.9300
C4—C9	1.404 (2)	C12—H12	0.9300
C5—C6	1.381 (3)	C14—H14	0.9300
C6—C7	1.370 (3)	C15—H15	0.9300
C7—C8	1.377 (3)		
			
C2—O4—C10	116.71 (11)	C10—C11—C12	119.42 (18)
C1—N1—C3	96.38 (13)	C11—C12—C13	119.89 (18)
O2—N2—O3	122.79 (14)	Cl1—C13—C14	119.24 (15)
O2—N2—C9	118.87 (13)	Cl1—C13—C12	119.92 (14)
O3—N2—C9	118.34 (13)	C12—C13—C14	120.82 (17)
O1—C1—N1	132.86 (15)	C13—C14—C15	119.57 (17)
O1—C1—C2	135.24 (15)	C10—C15—C14	120.02 (16)
N1—C1—C2	91.90 (12)	O4—C2—H2	112.00
C1—N1—H1	132.00	C1—C2—H2	112.00
C3—N1—H1	132.00	C3—C2—H2	112.00
O4—C2—C1	118.81 (12)	N1—C3—H3	112.00
O4—C2—C3	114.90 (12)	C2—C3—H3	112.00
C1—C2—C3	85.17 (11)	C4—C3—H3	112.00
N1—C3—C2	85.85 (11)	C4—C5—H5	119.00
C2—C3—C4	116.85 (14)	C6—C5—H5	119.00
N1—C3—C4	114.34 (14)	C5—C6—H6	120.00
C3—C4—C9	123.94 (14)	C7—C6—H6	120.00
C3—C4—C5	120.12 (14)	C6—C7—H7	120.00
C5—C4—C9	115.94 (14)	C8—C7—H7	120.00
C4—C5—C6	121.75 (16)	C7—C8—H8	120.00
C5—C6—C7	120.68 (18)	C9—C8—H8	120.00
C6—C7—C8	119.63 (18)	C10—C11—H11	120.00
C7—C8—C9	119.33 (16)	C12—C11—H11	120.00
N2—C9—C8	116.69 (14)	C11—C12—H12	120.00
C4—C9—C8	122.66 (15)	C13—C12—H12	120.00
N2—C9—C4	120.65 (13)	C13—C14—H14	120.00
C11—C10—C15	120.23 (15)	C15—C14—H14	120.00
O4—C10—C11	123.40 (14)	C10—C15—H15	120.00
O4—C10—C15	116.34 (13)	C14—C15—H15	120.00
			
C10—O4—C2—C3	156.31 (12)	C2—C3—C4—C9	−70.06 (19)
C2—O4—C10—C11	32.5 (2)	C3—C4—C9—N2	1.0 (2)
C2—O4—C10—C15	−149.62 (14)	C3—C4—C9—C8	−179.81 (15)
C10—O4—C2—C1	−105.11 (15)	C3—C4—C5—C6	179.68 (17)
C3—N1—C1—C2	6.71 (14)	C9—C4—C5—C6	−1.2 (2)
C3—N1—C1—O1	−173.8 (2)	C5—C4—C9—N2	−178.08 (14)
C1—N1—C3—C2	−6.60 (14)	C5—C4—C9—C8	1.1 (2)
C1—N1—C3—C4	111.06 (16)	C4—C5—C6—C7	0.3 (3)
O3—N2—C9—C4	158.62 (14)	C5—C6—C7—C8	0.8 (3)
O2—N2—C9—C8	158.71 (15)	C6—C7—C8—C9	−0.9 (3)
O3—N2—C9—C8	−20.7 (2)	C7—C8—C9—C4	−0.1 (2)
O2—N2—C9—C4	−22.0 (2)	C7—C8—C9—N2	179.17 (15)
N1—C1—C2—C3	−6.30 (14)	O4—C10—C11—C12	178.78 (18)
N1—C1—C2—O4	−122.13 (15)	C15—C10—C11—C12	1.0 (3)
O1—C1—C2—O4	58.4 (3)	O4—C10—C15—C14	−177.37 (15)
O1—C1—C2—C3	174.2 (2)	C11—C10—C15—C14	0.6 (3)
O4—C2—C3—N1	125.34 (13)	C10—C11—C12—C13	−2.4 (3)
C1—C2—C3—N1	5.73 (12)	C11—C12—C13—Cl1	−179.60 (18)
C1—C2—C3—C4	−109.51 (14)	C11—C12—C13—C14	2.2 (4)
O4—C2—C3—C4	10.09 (19)	Cl1—C13—C14—C15	−178.84 (15)
N1—C3—C4—C9	−168.11 (14)	C12—C13—C14—C15	−0.6 (3)
C2—C3—C4—C5	108.95 (17)	C13—C14—C15—C10	−0.8 (3)
N1—C3—C4—C5	10.9 (2)		

### Hydrogen-bond geometry (Å, º)

Cg2 is the centroid of the nitrobenzene ring (C4–C9).

**Table:**

*D*—H···*A*	*D*—H	H···*A*	*D*···*A*	*D*—H···*A*
N1—H1···O1^i^	0.86	2.09	2.936 (2)	166
C8—H8···O3^ii^	0.93	2.53	3.307 (2)	142
C15—H15···O2^iii^	0.93	2.51	3.338 (2)	149
C3—H3···*Cg*2^iv^	0.98	2.70	3.5118 (19)	141

Symmetry codes: (i) −*x*+1/2, *y*+1/2, −*z*+3/2; (ii) −*x*+1, −*y*, −*z*+1; (iii) *x*, *y*−1, *z*; (iv) *x*, *y*+1, *z*.
