# Supplementary figures and images for: Crystal structure of 3-(4-chloro­phen­oxy)-4-(2-nitro­phen­yl)azetidin-2-one with an unknown solvate
Source: Acta Crystallogr E Crystallogr Commun. 2015 Jan 1;71(Pt 1):o8–9. doi: 10.1107/S2056989014025845 (PMC4331907; doi:10.1107/S2056989014025845)

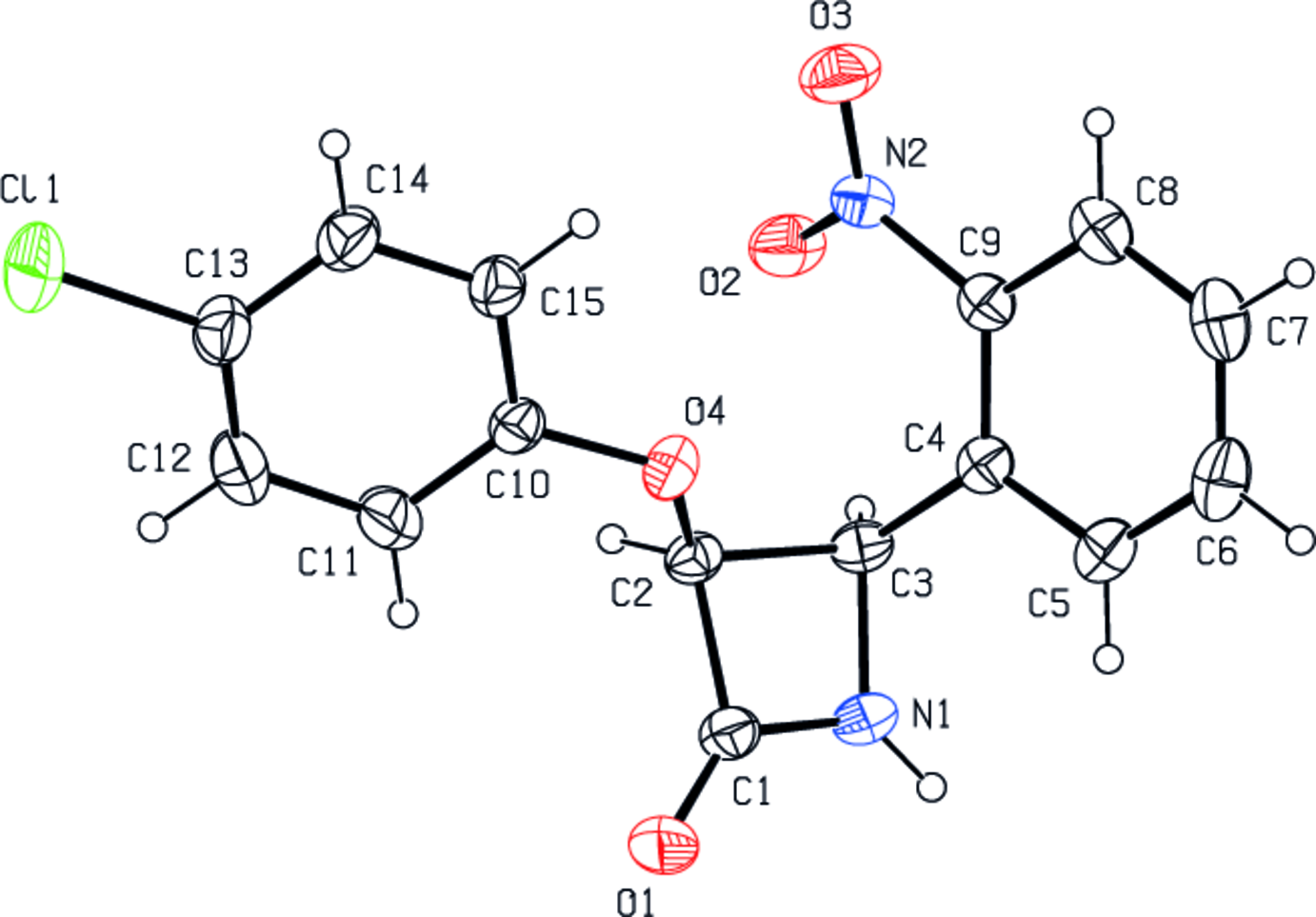

Supplement: Supplementary file 4 [file e-71-000o8-fig1.tif]

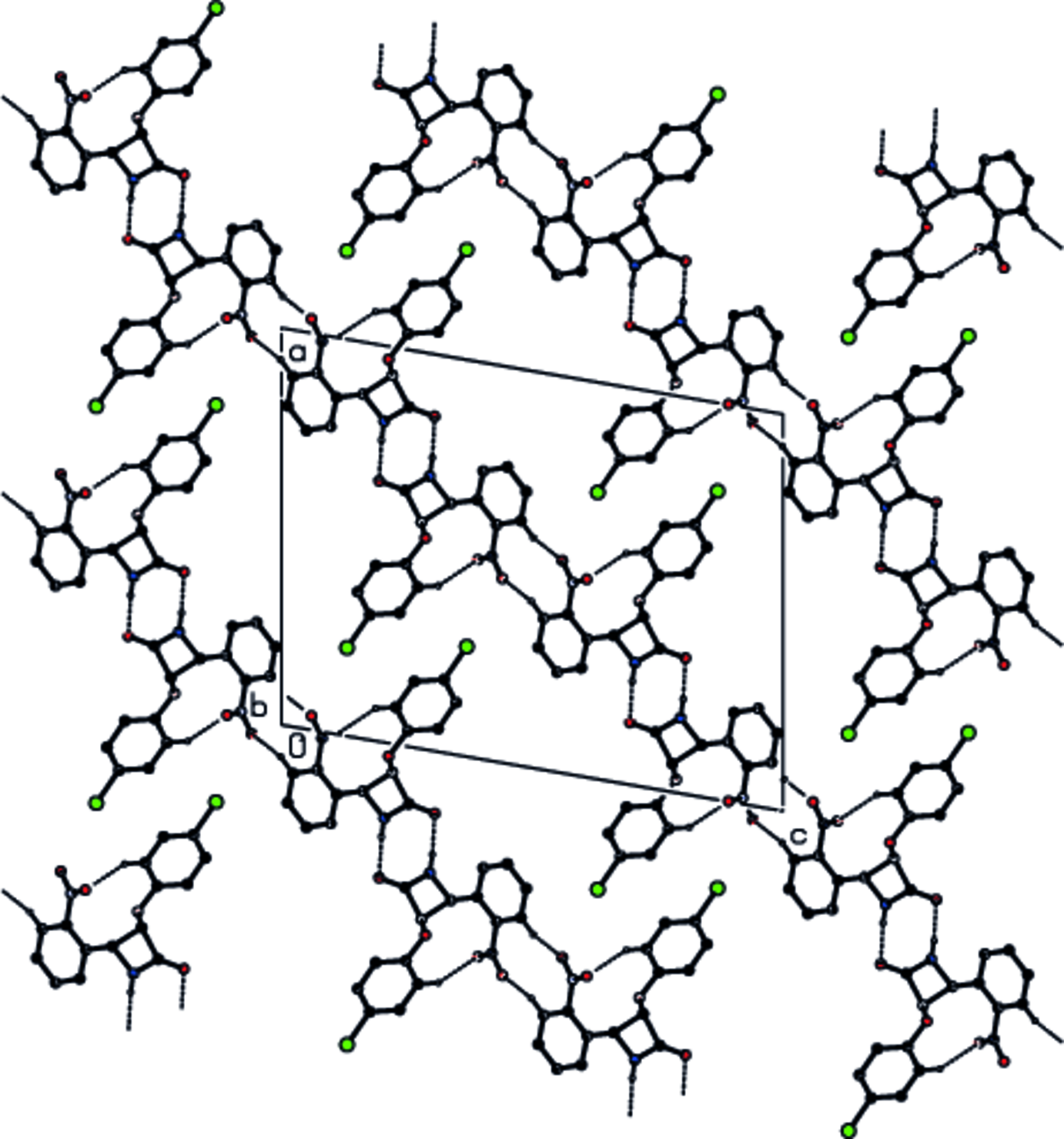

Supplement: Supplementary file 5 [file e-71-000o8-fig2.tif]
